# Supplementary material for: Unique structure and function of viral rhodopsins
Source: Nat Commun. 2019 Oct 30;10:4939. doi: 10.1038/s41467-019-12718-0 (PMC6821725; doi:10.1038/s41467-019-12718-0)
Supplement: Supplementary file 1 — Supplementary Information [file 41467_2019_12718_MOESM1_ESM.pdf]

Supplementary Information for
"Unique structure and function of viral rhodopsins"

D. Bratanov *et al.*

Supplementary Figures

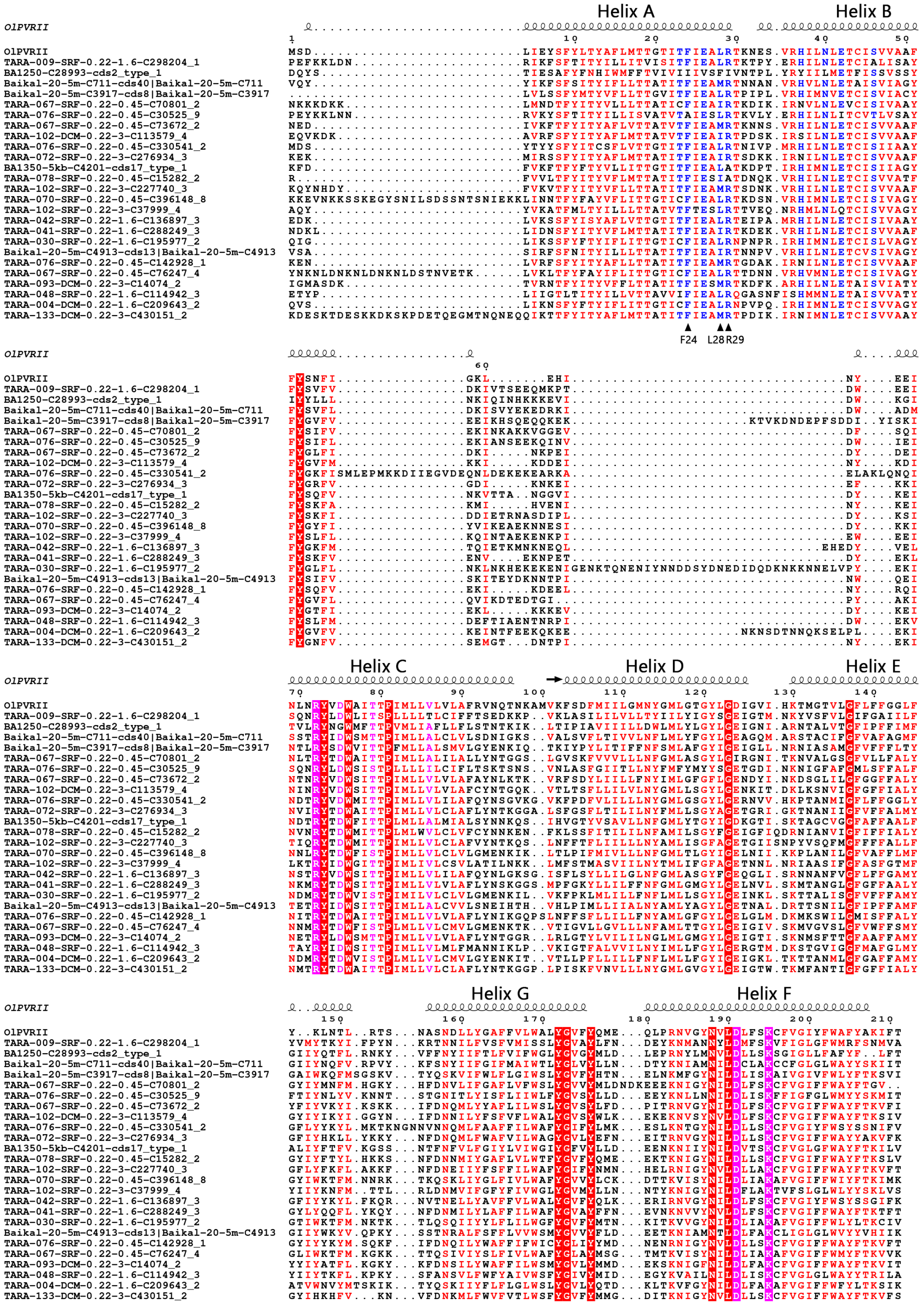

Supplementary Fig. 1 Sequence alignment of viral rhodopsins from group II including the representative TARA metagenomic sequences. Highly conservative residues are indicated with red

color, 6-letter motif residues are indicated with magenta color. Residues involved into formation of the putative channel pore are colored blue.

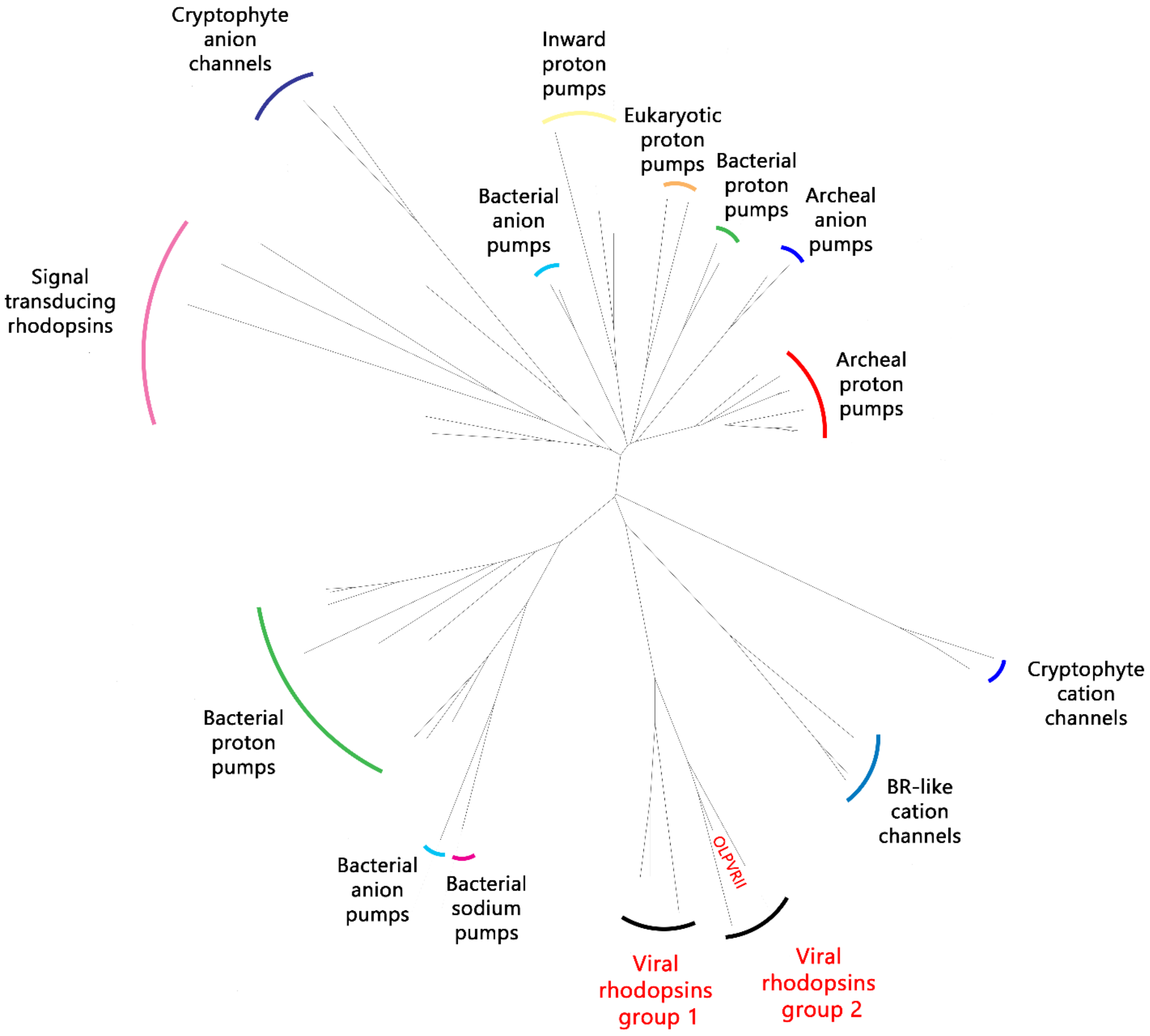

Supplementary Fig. 2 A phylogenetic tree of microbial rhodopsins. Different microbial rhodopsins groups and their functions are indicated. Viral rhodopsins and OLPVRII are highlighted red.

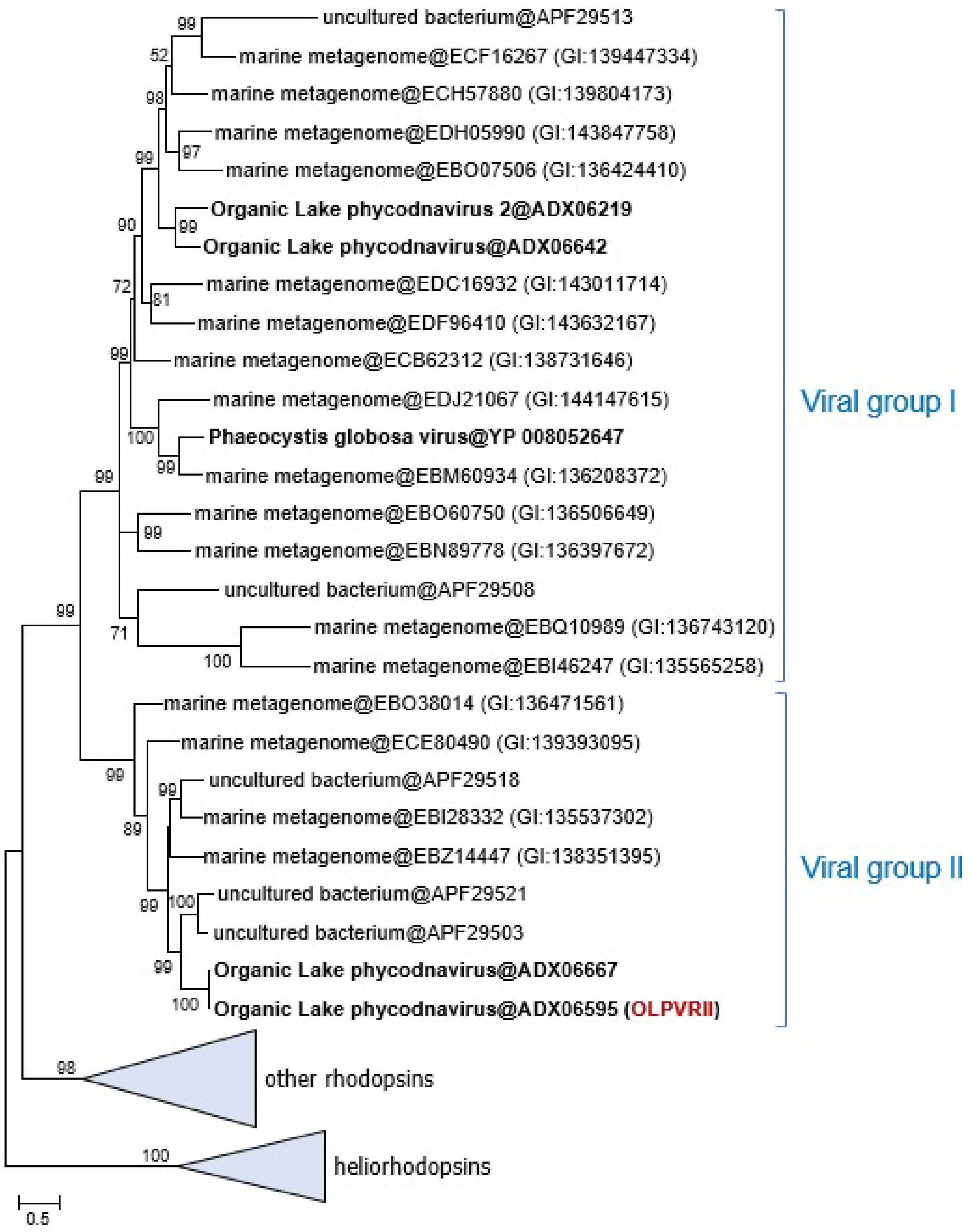

Supplementary Fig. 3 A phylogenetic tree of the viral rhodopsin superfamily including already published sequences.

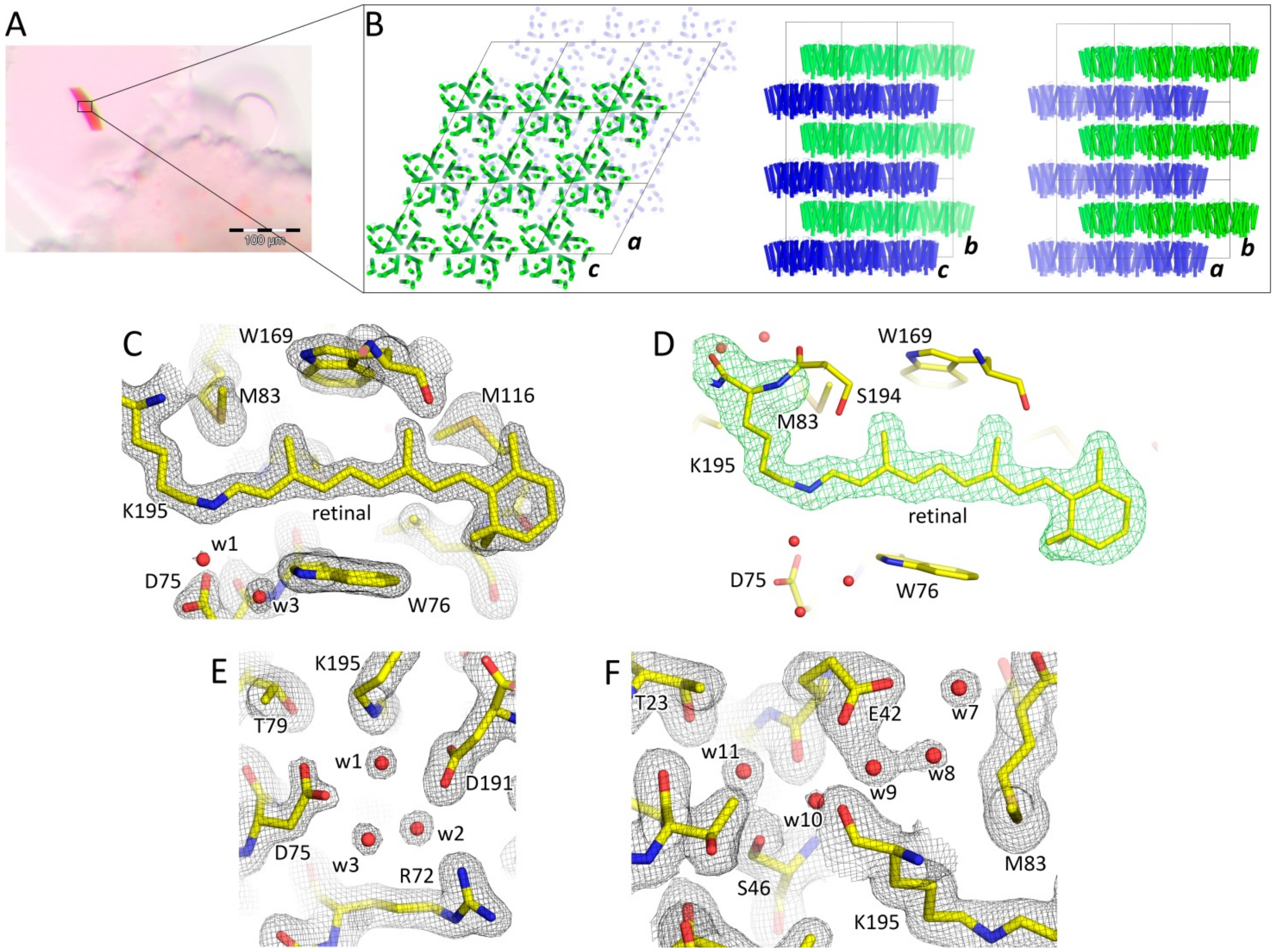

Supplementary Fig. 4 Crystal packing and examples of 2Fo-Fc electron density maps. **a** Example of OLPVRII crystals. **b** Crystal packing of OLPVRII. **c** Example of 2Fo-Fc electron density maps around retinal of protomer B. Maps a contoured at the level of 1.5σ. **d** Simulated annealing omitted electron density map built omitting the retinal and Lys195 residues. The map is contoured at the level of 3.0 σ. **e** Example of 2Fo-Fc electron density maps in the RSB region of protomer E. Maps a contoured at the level of 1.5σ. **f** Example of 2Fo-Fc electron density maps around Glu42 of protomer B. Maps a contoured at the level of 1.0σ.

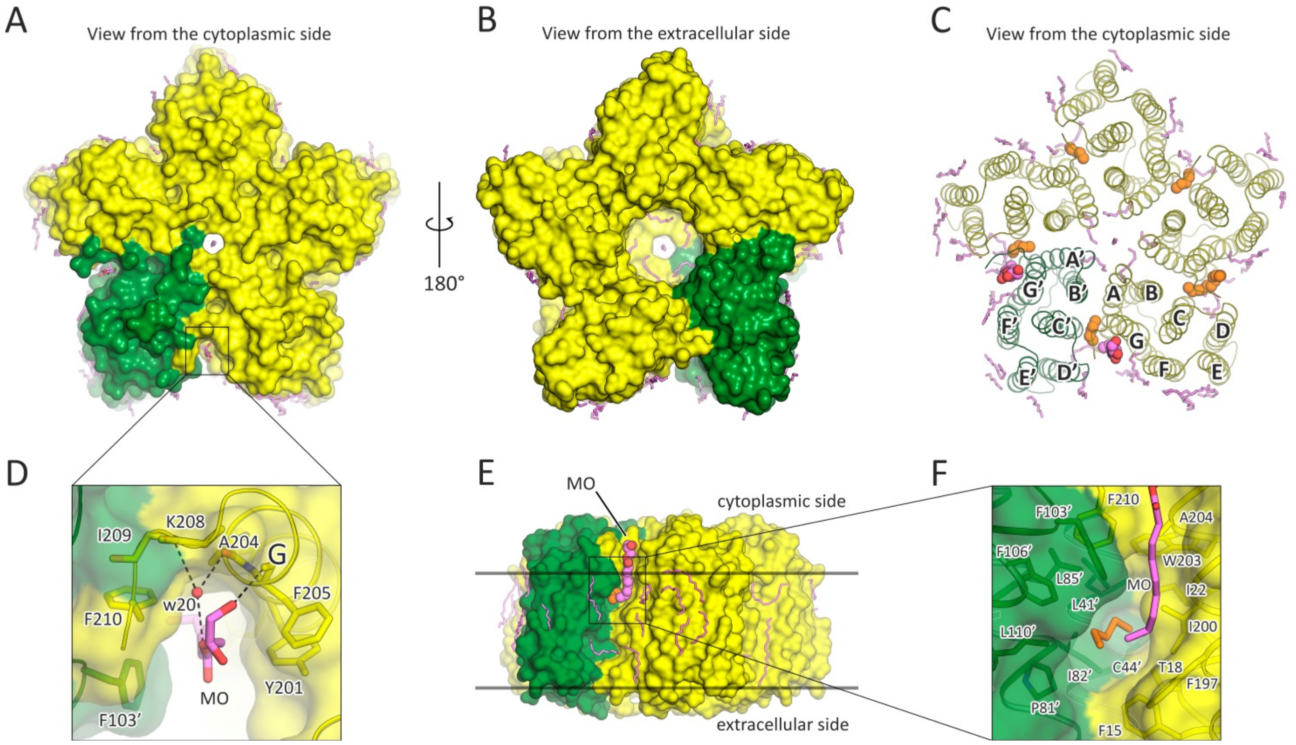

Supplementary Fig. 5 OLPVRII pentamer and lipids paving the pentamer. a View from the cytoplasmic side. **b** View from the extracellular side. Lipid fragments are colored violet. **c** View from the cytoplasmic side on the cartoon representation of the pentamer with surrounding lipids. Monoolein (MO) molecules are shown with spheres. Lipidic fragments deepened between the protomers (helices A and G from one protomer and B', C' and D' of the neighbouring protomer) are colored orange and shown with spheres. **d** Detail view of the MO stabilization hydrogen bonds. **e** Side view of the pentamer. Monoolein (MO) molecule is shown with spheres. Lipidic fragment deepened between the protomers is colored orange and shown with spheres. Hydrophobic/hydrophilic borders of the membrane are shown with gray lines. **f** Detail side view of the residues comprising pocket for lipidic molecule deepened between protomers. One protomer is colored green.

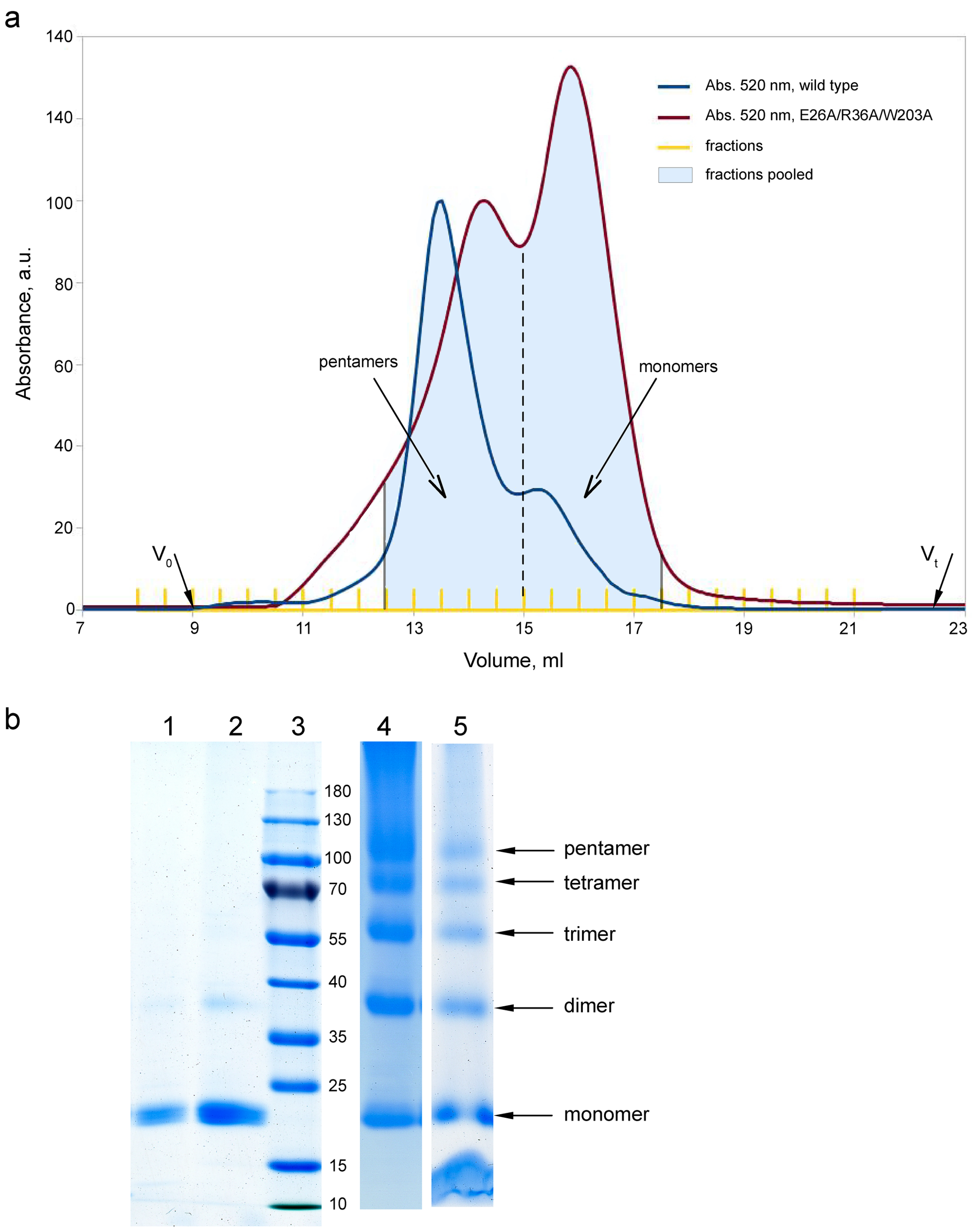

Supplementary Fig. 6 Investigation of the OLPVRII pentamerization. **a** Typical SEC profile of wild type OLPVRII is shown in blue. The elution profile of E26A/R36A/W203A mutant is shown in red. The fractions pooled are indicated, as well as distribution between monomers and pentamers.

We suppose that the relative shifts of the monomer and pentamer peaks are probably caused by a combination of several factors: different columns used, peak overlapping, influence of the mutation itself, difference of the detergent binding to the wild type and mutant proteins. Nevertheless the elution profiles clearly illustrate the distribution of the protein between monomeric and pentameric fractions. **b** SDS-PAGE analysis of SEC fractions and results of crosslinking experiments. Lane 1: Monomeric fraction after gel-filtration; lane 2: Pentameric fraction after gel-filtration; lane 3: molecular weight marker with molecular weights indicated; lane 4: pentameric protein at 2 mg/ml protein, crosslinked with 10% glutaraldehyde, 8 hours incubation; lane 5: monomeric protein at 1 mg/ml reconstituted into POPC/POPG mixture crosslinked with 10% glutaraldehyde, 8 hours incubation. Source data are provided as a Source Data file.

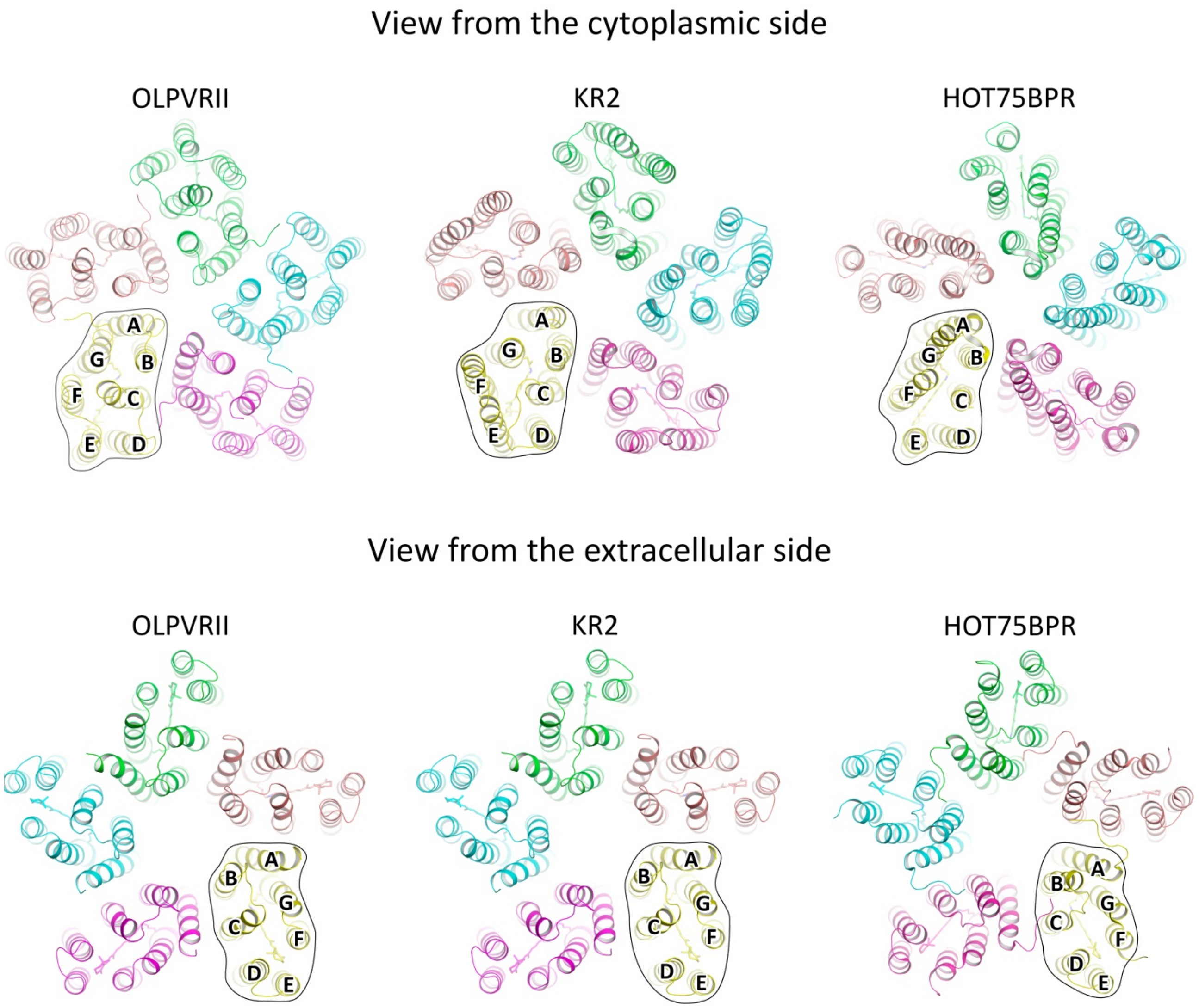

Supplementary Fig. 7 Architecture of the pentamers of microbial rhodopsins.

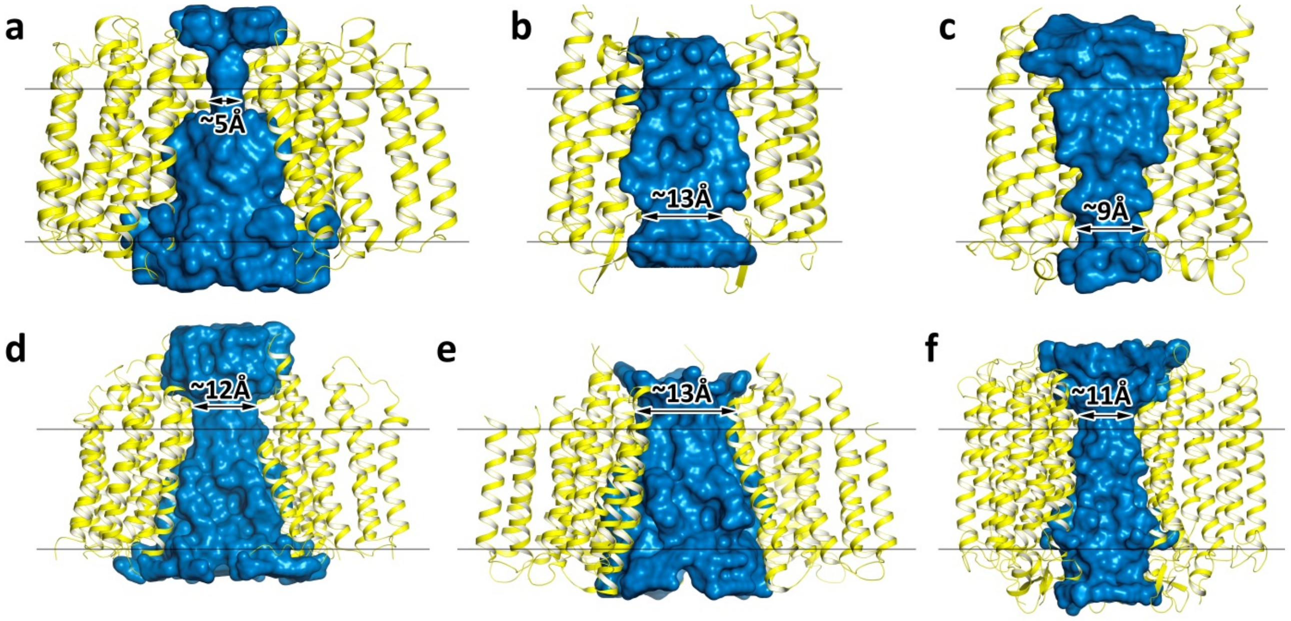

Supplementary Fig. 8 Central part of OLPVRII and other rhodopsins. **a** OLPVRII. **b** bR (PDB ID 1C3W). **c** NsXeR (PDB ID 6EYU). **d** BPR HOT75 (PDB ID 4KLY). **e** BPR Med12 (PDB ID 4JQ6). **f** KR2 (PDB ID 4XTO). Cavities are shown in blue. One protomer of each oligomer is hidden for clarity.

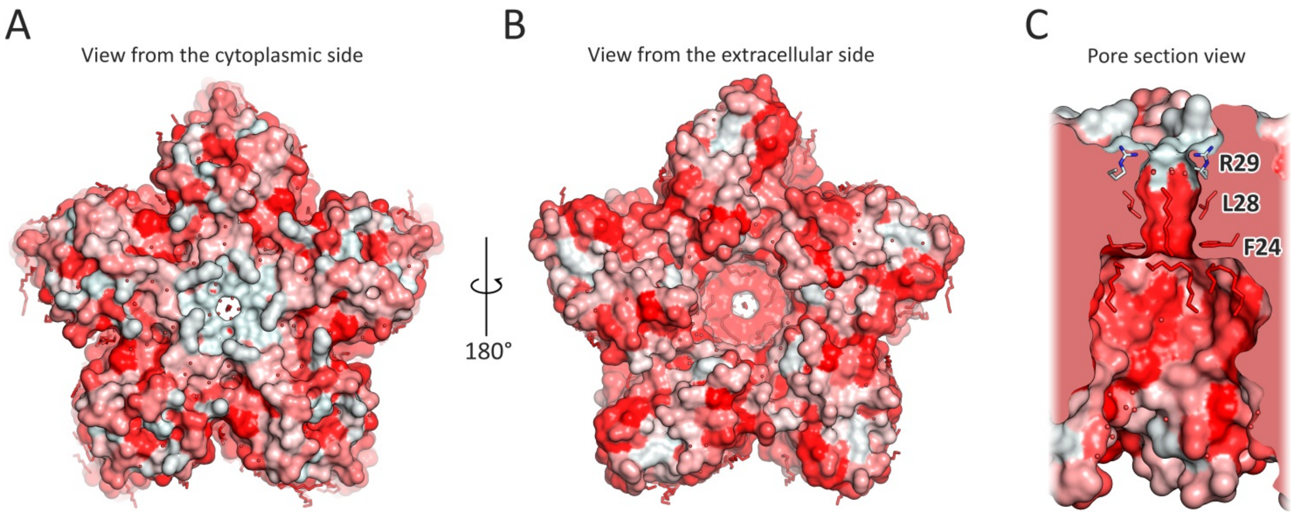

Supplementary Fig 9 Hydrophobicity of the OLPVRII pentamer surface. **a** View from the cytoplasmic side. **b** View from the extracellular side. **c** Side section view of the central pore. Residues forming the pore vestibule are shown with sticks (Arg29, Leu28 and Phe24). Lipid fragments are shown with sticks and are colored red. Water molecules are shown with red spheres. Red color of the residue indicates its hydrophobicity, while white color indicates its hydrophilicity.

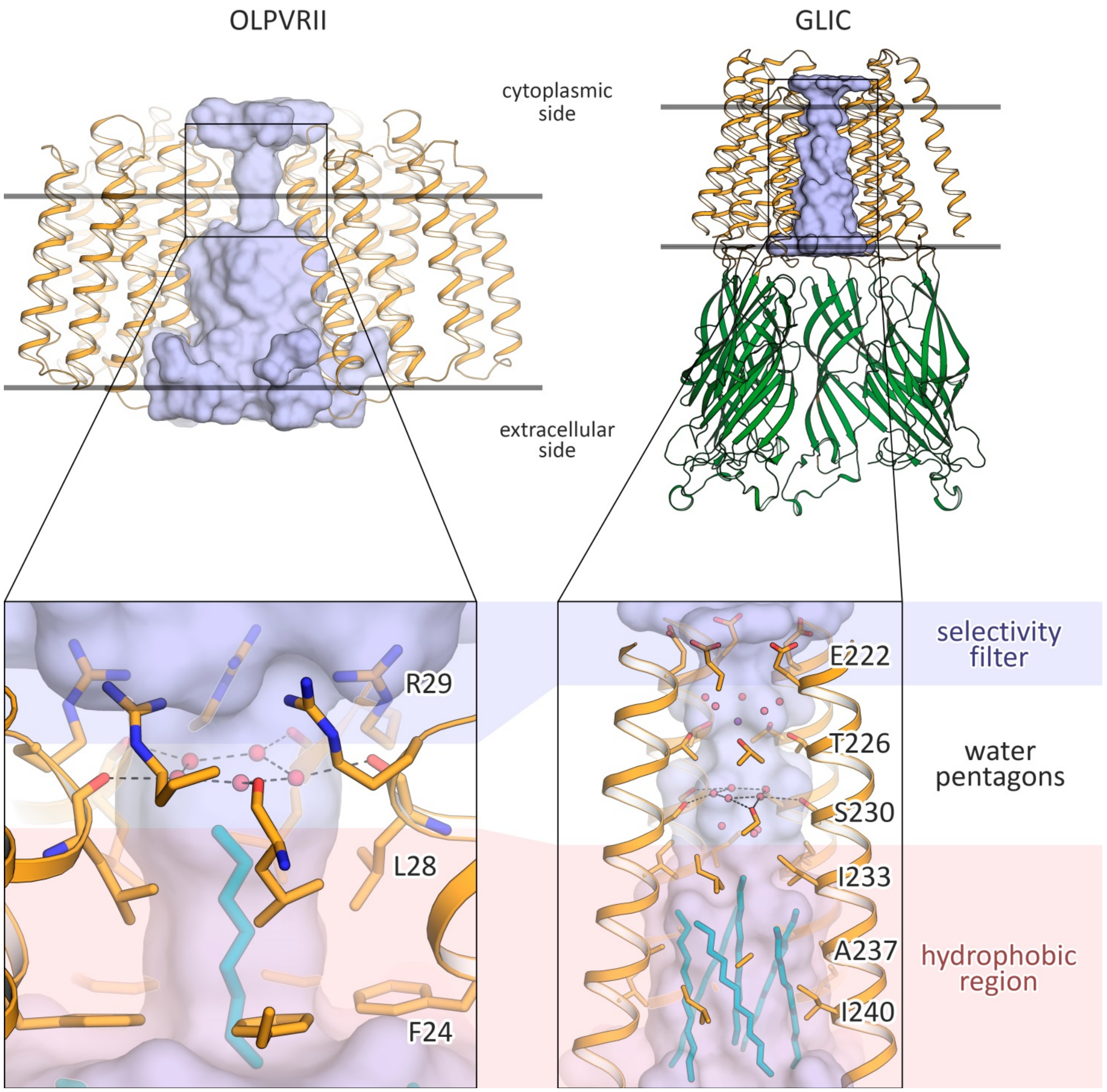

Supplementary Fig. 10 Comparison of the inner pore of OLPVRII (left) and GLIC (right). For GLIC, PDB ID: 4HFI was used. Extracellular domain is colored green. Hydrocarbon chains are colored cyan.

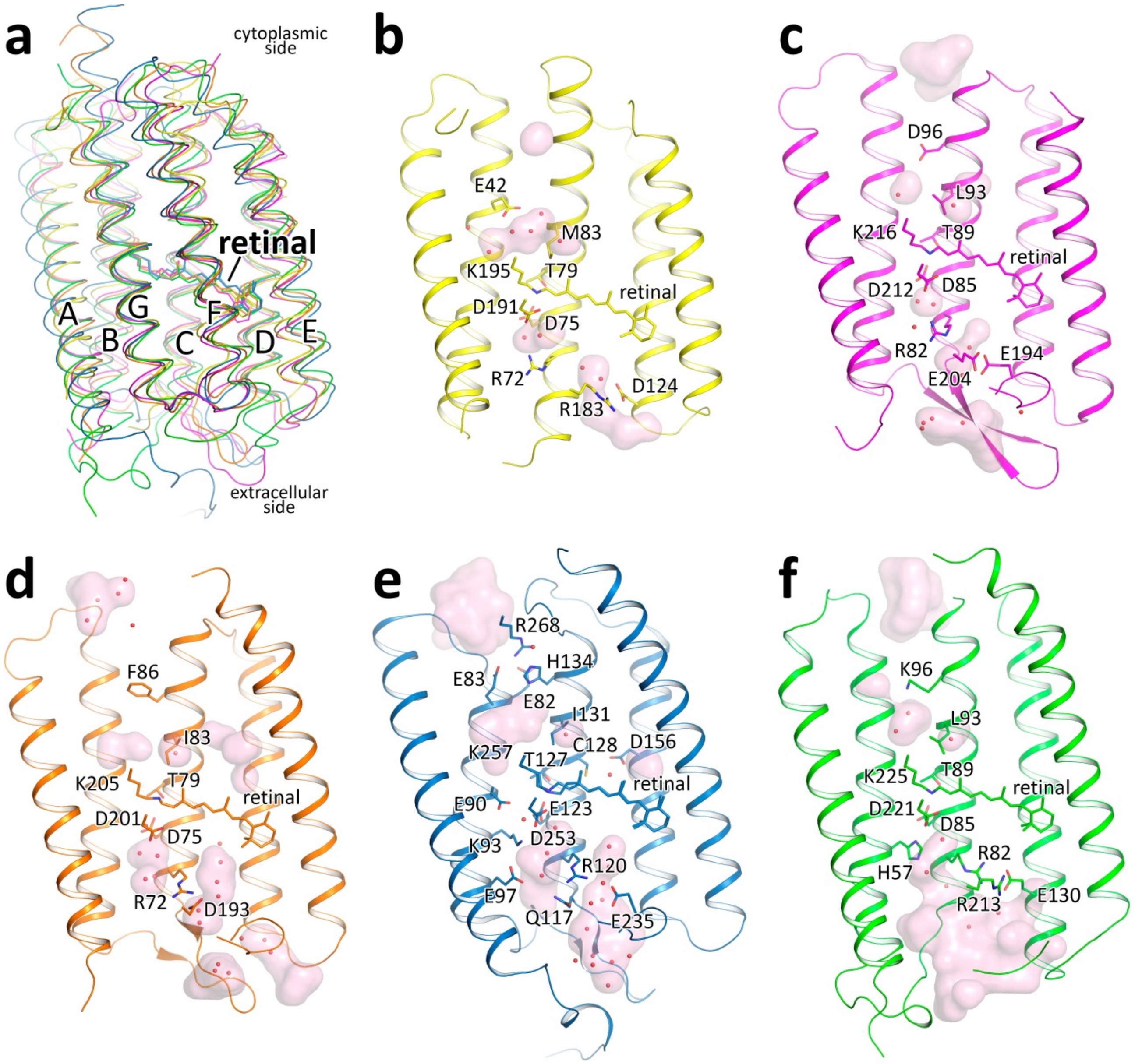

Supplementary Fig. 11 Comparison of OLPVRII with other rhodopsins. **a** Structural alignment with OLPVRII (yellow), bR (purple), NpSRII (orange), ESR (green) and ChR2 (blue). The most different is location of helices A and B. **b** Side view of OLPVRII. **c** Side view of bR (PDB ID 1C3W). **d** Side view of NpSRII (PDB ID 1H2S). **e** Side view of ChR2 (PDB ID 6EID). **f** Side view of ESR (PDB ID 4HYJ). Cavities inside protein protomers are colored pink. Key residues are shown with sticks. Helices F and G are hidden for clarity.

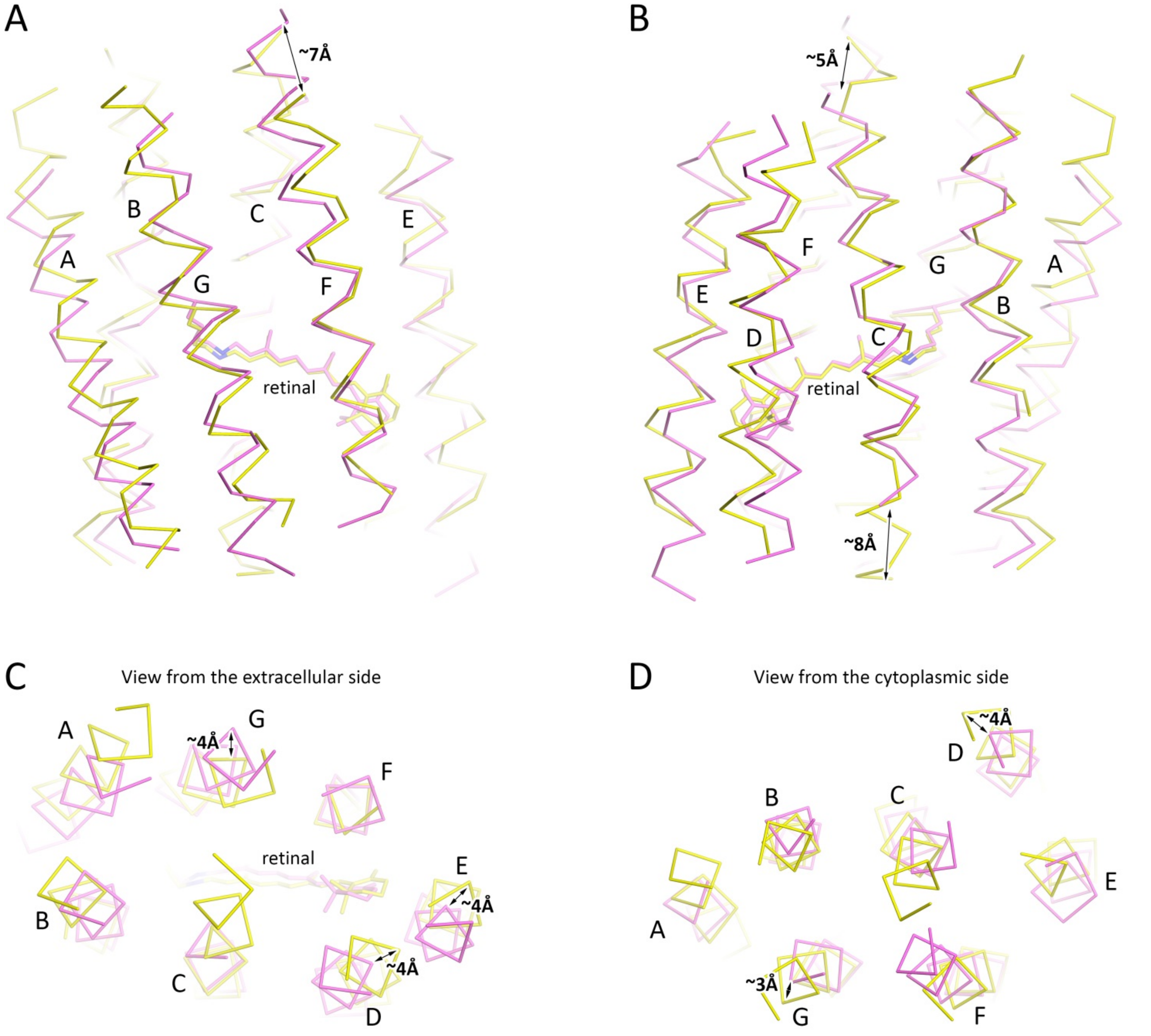

Supplementary Fig 12 Structural alignment of OLPVRII (yellow) and bR (magenta) protomers. **a, b** Side views. **c, d** View from the extracellular and cytoplasmic sides, respectively. The most notable differences are marked with black arrows.

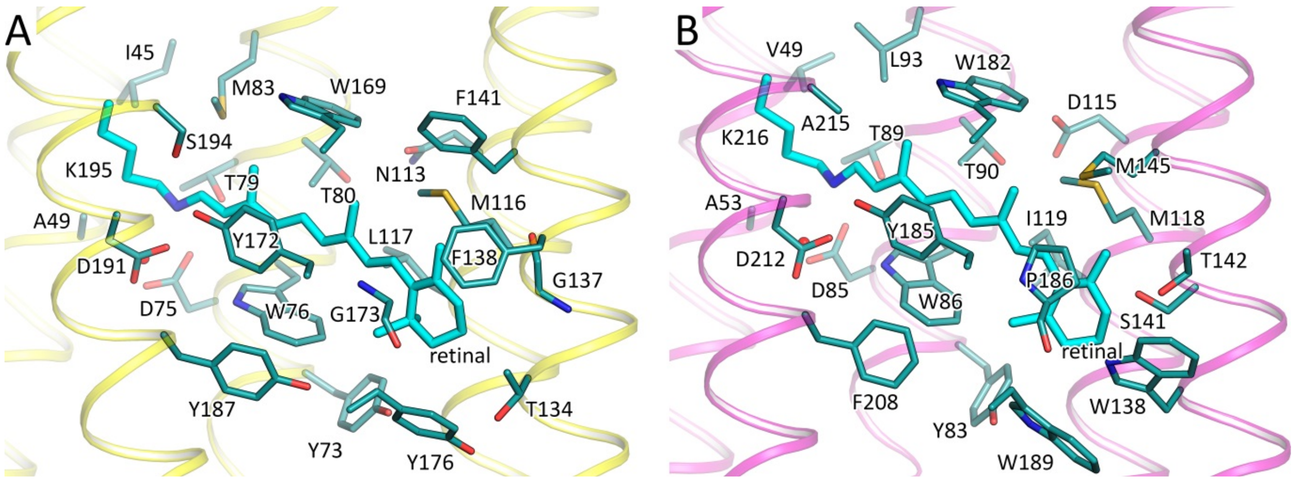

Supplementary Fig. 13 Retinal binding pocket of a OLPVRII; **b** bR (PDB ID: 1C3W). Residues comprising retinal binding pocket are colored teal, retinal molecules are colored cyan.

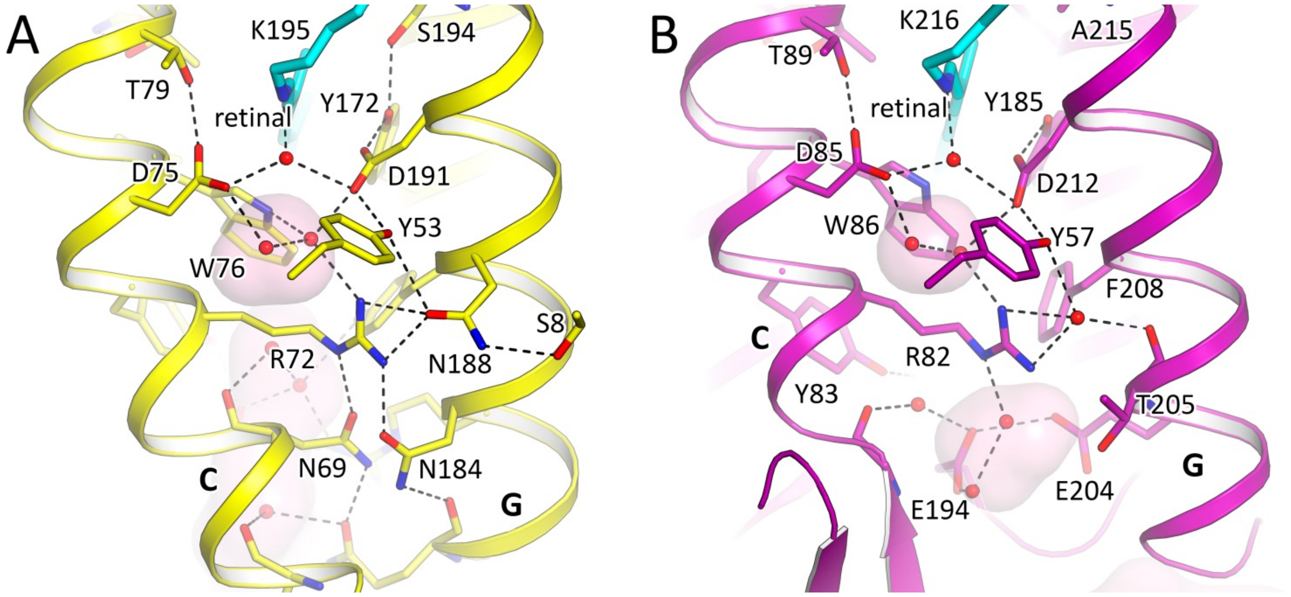

Supplementary Fig. 14 Extracellular part of the Schiff base region of **a** OLPVRII; **b** bR (PDB ID: 1C3W). Retinal molecules are colored cyan, cavities are colored pink. Helices and C and G are annotated with capital bold letters. Cavities inside the protein protomers are colored pink.

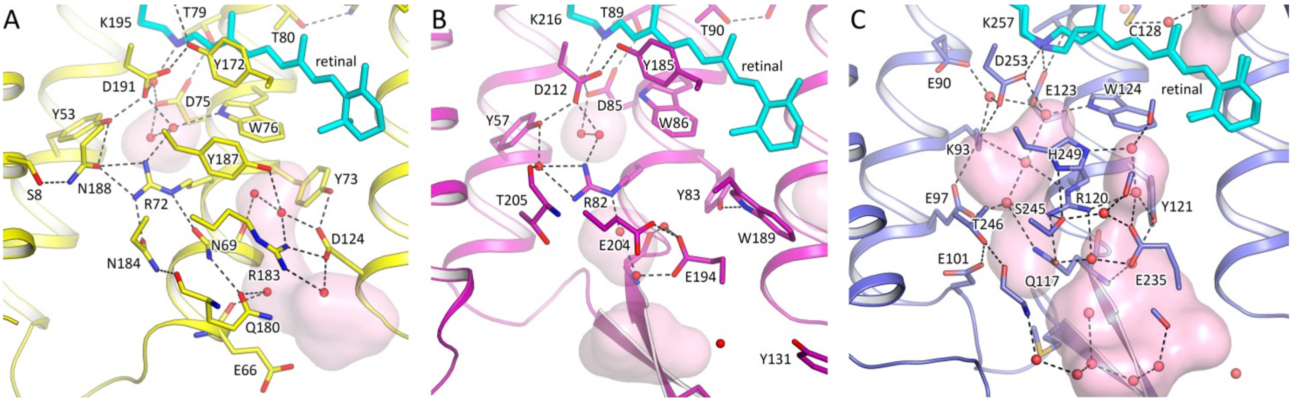

Supplementary Fig. 15 Extracellular part of **a** OLPVRII; **b** bR (PDB ID: 1C3W), **c** ChR2 (PDB ID: 6EID). Retinal molecules are colored cyan, cavities inside protein protomers are colored pink.

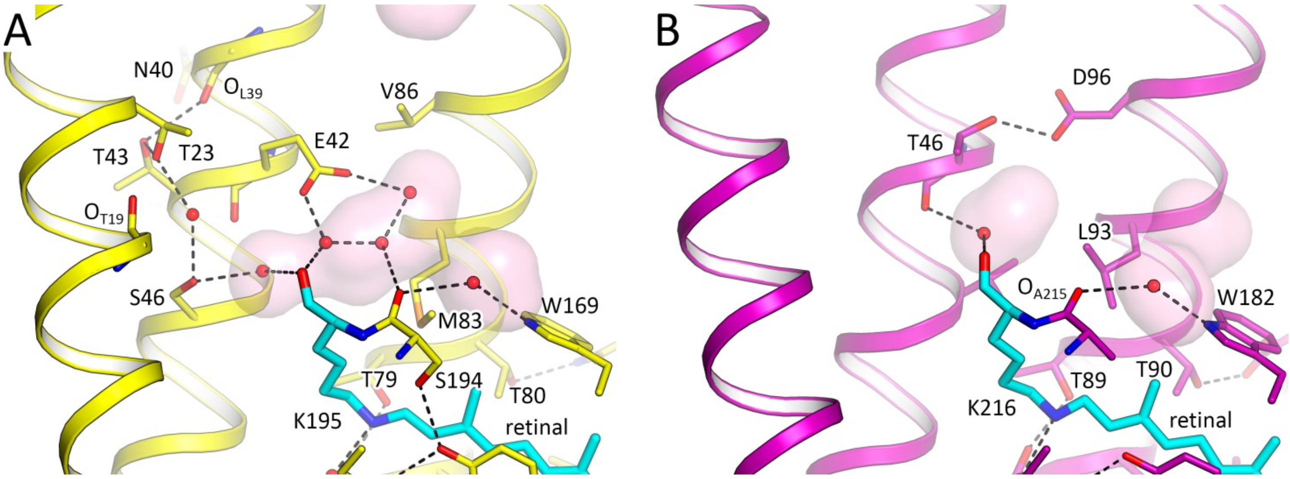

Supplementary Fig. 16 Cytoplasmic part of **a** OLPVRII; **b** bR (PDB ID: 1C3W). Retinal molecules are colored cyan, cavities inside protein protomers are colored pink. Helices A, B and C are annotated with capital bold letters.

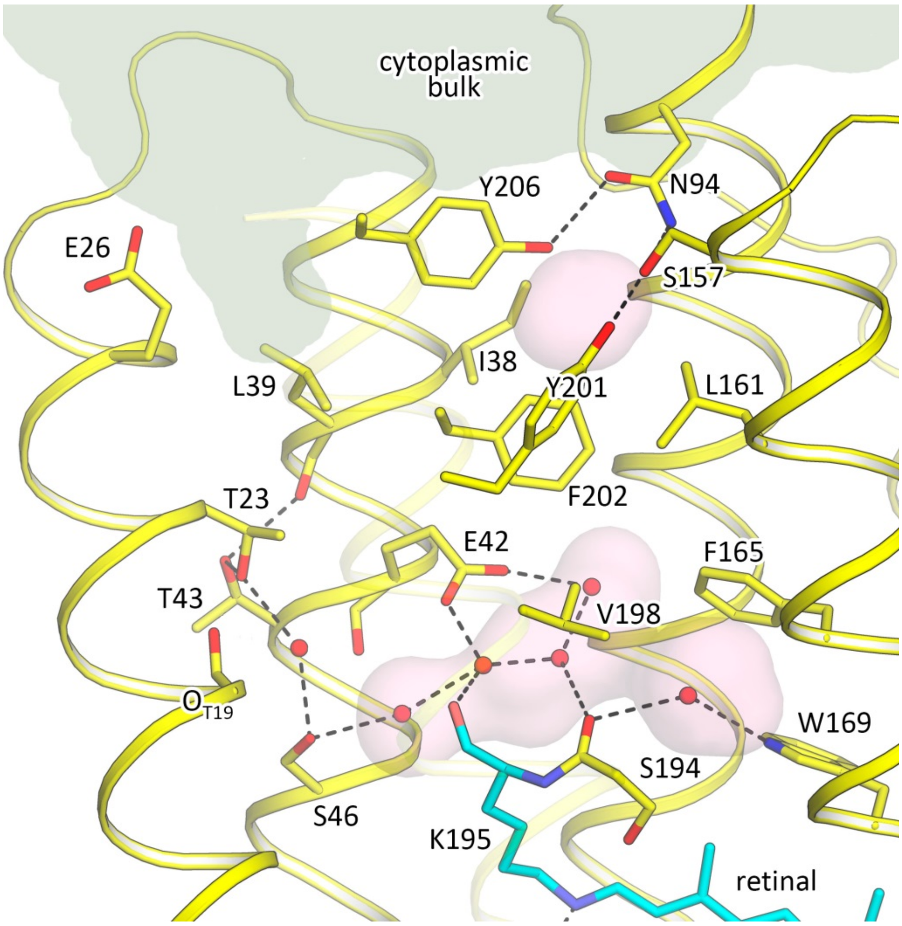

Supplementary Fig. 17 Cytoplasmic part of OLPVII. Gray color indicates the cytoplasmic space, which concaves inside the protein down to Leu39 side chain. Cavities inside the protein protomer are colored pink.

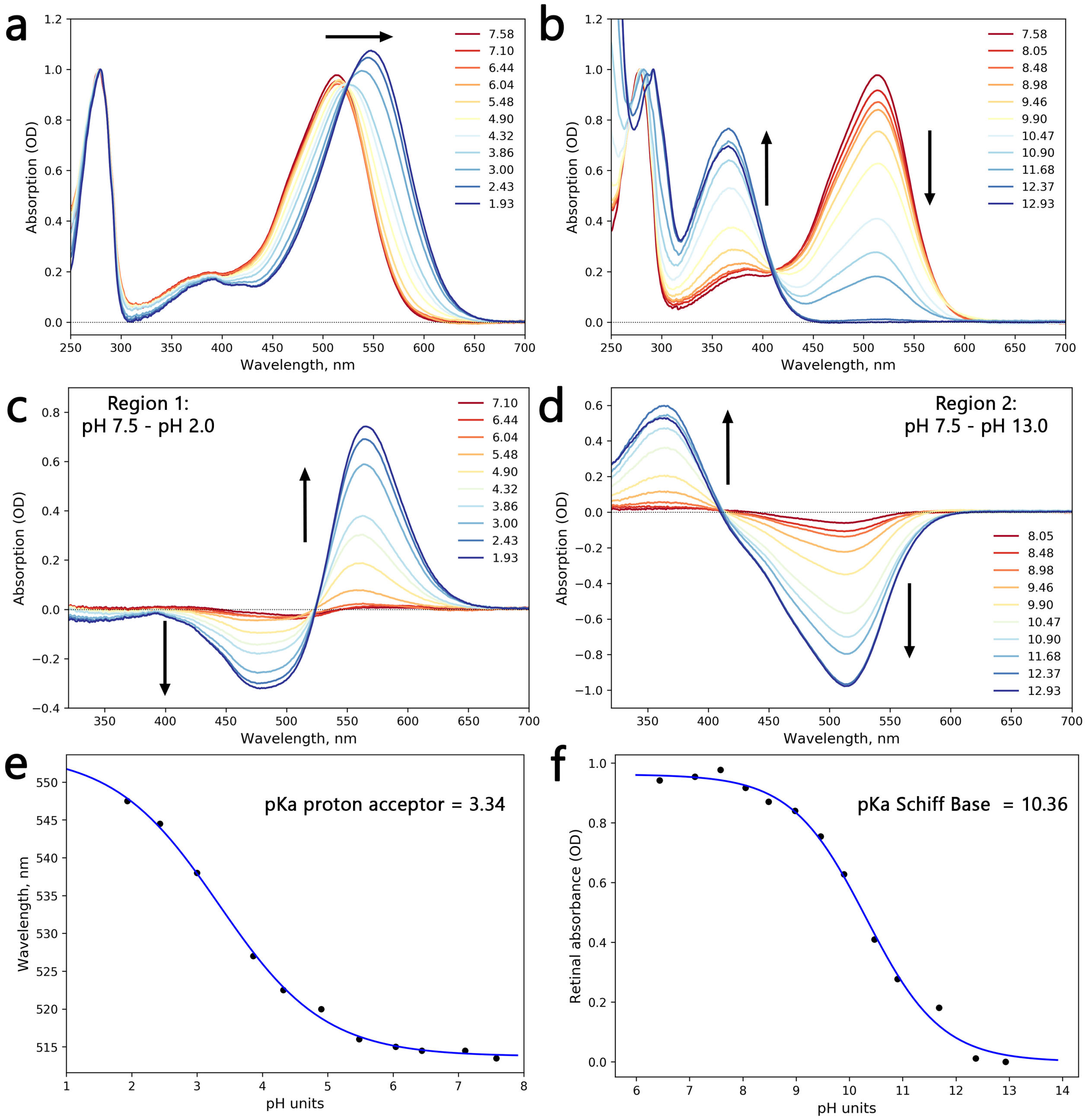

Supplementary Fig. 18 pH titration of solubilized OLPVRII. **a** Absorbtion spectra at pH between 7.58 and 12.93. **b** Absorbtion spectra at pH between 7.58 and 1.93. **c** Difference spectra at pH from 8.05 to 12.93. **d** Difference spectra at pH from 8.05 to 1.93. **e** Fitting at lower pH values of the dependence of the retinal absorbance maximum on the pH value with Boltzmann sigmoidal curve. The the point of half decay of the fit gave the pKa of the proton acceptor ± standard error. **f** Fitting at higher pH values of dependence of the absorbance at 514 nm on the pH value with Boltzmann sigmoidal curve. The point of half decay of the fit gave the pKa of the Schiff base ± standard error. Source data are provided as a Source Data file.

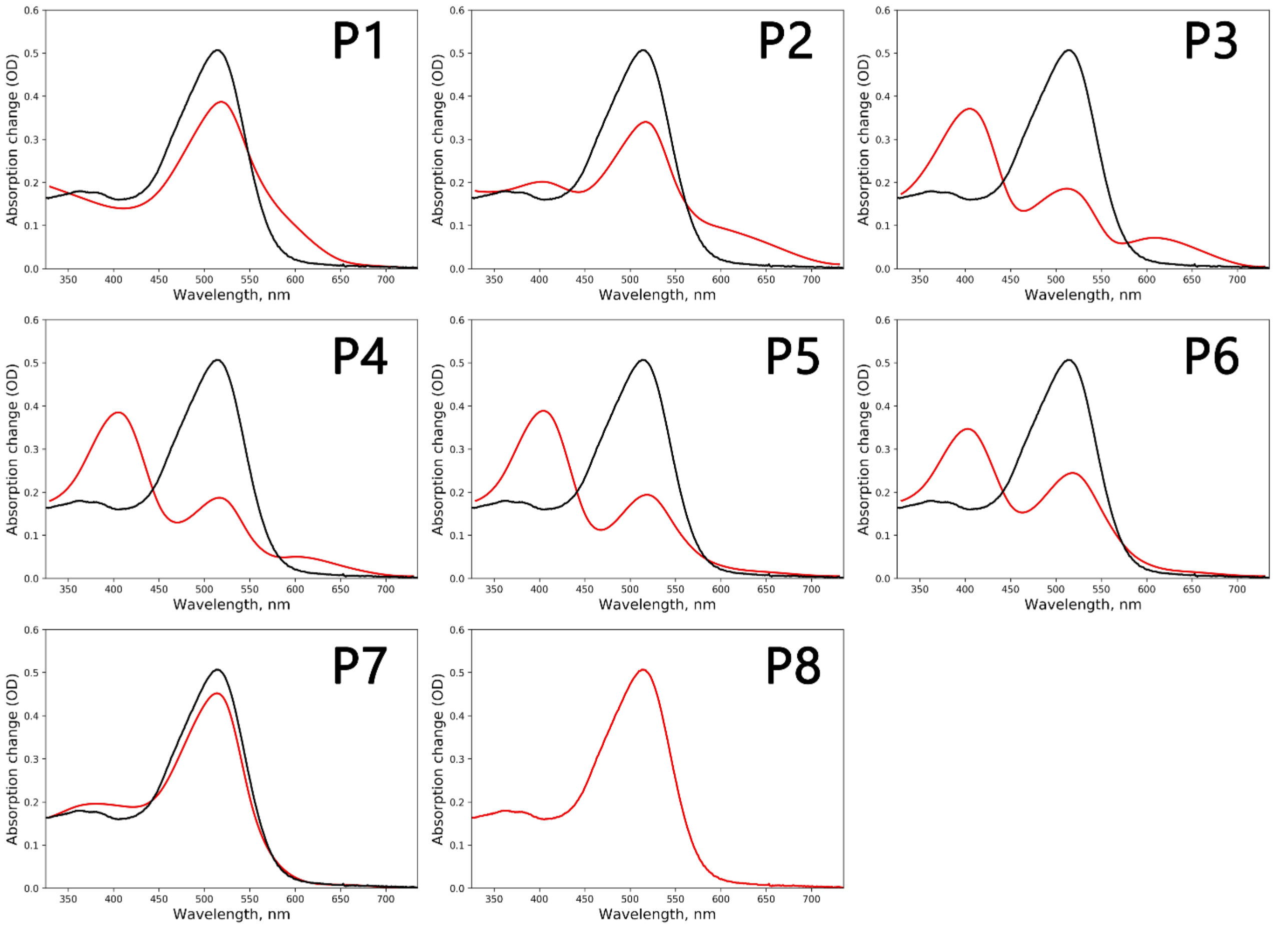

Supplementary Fig. 19 Intermediate states spectra of OLPVRII. The absolute spectra of 7 intermediate state determined during transient kinetics data treatments are shown with red color, while ground state spectra is represented with black line. Source data are provided as a Source Data file.

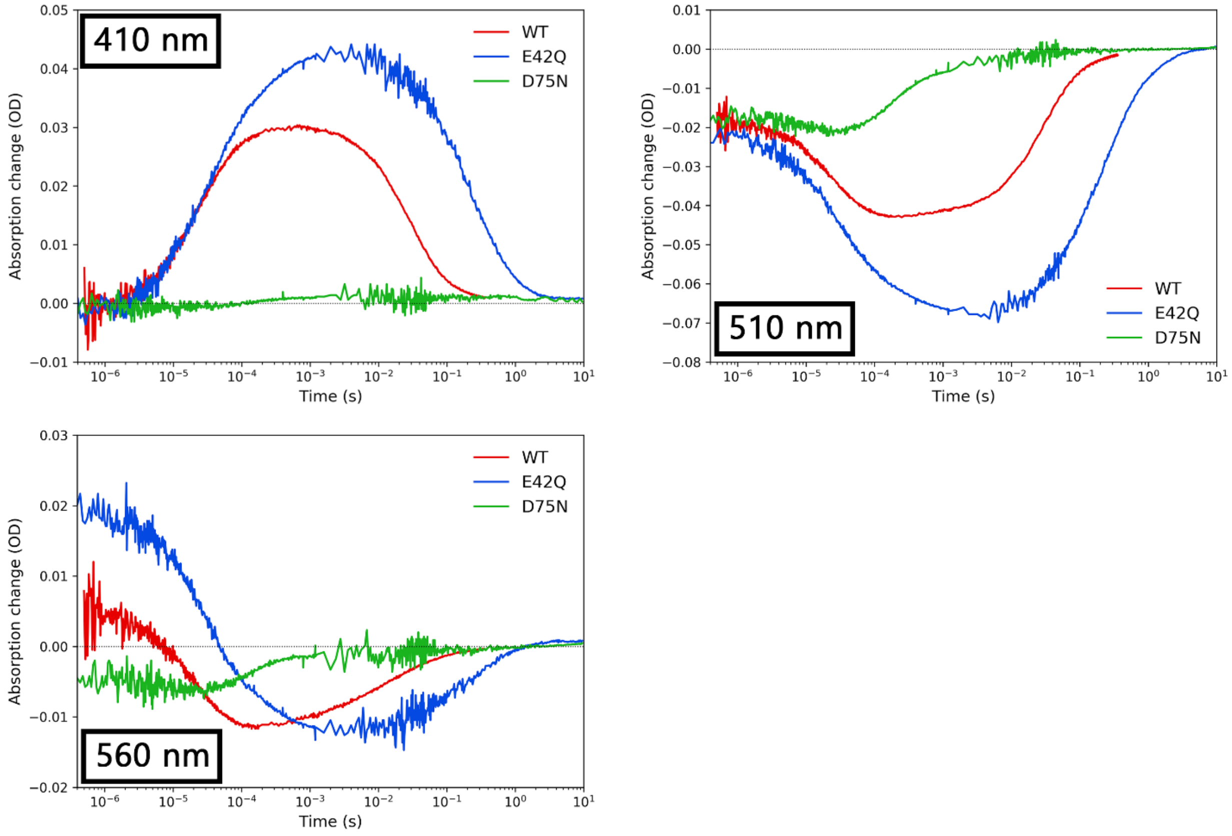

Supplementary Fig. 20 Photocycle kinetics of OLPVRII mutants. Time dependence of absorption differences at selected wavelengths of the wild type OLPVRII and its mutants E42Q and D75N colored red, blue and green, respectively. Source data are provided as a Source Data file.

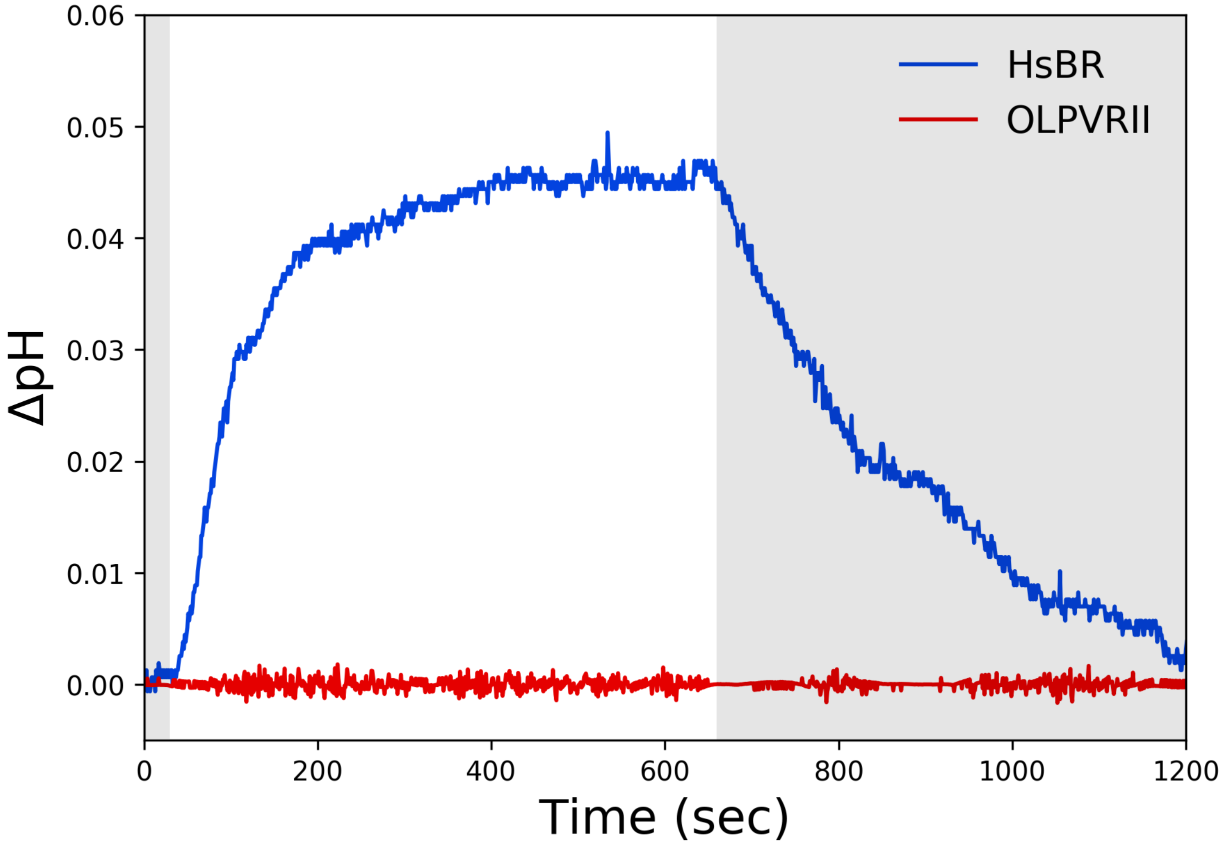

Supplementary Fig. 21 Pumping activity of OLPVRII reconstituted into liposomes. Representative examples of pH change of the suspension after illumination of HsbR (blue) and OLPVRII (red) proteins introduced into liposomes. The illumination onset is indicated by gray (light off) and white (light on) colors. Experimental conditions are directly comparable. Source data are provided as a Source Data file.

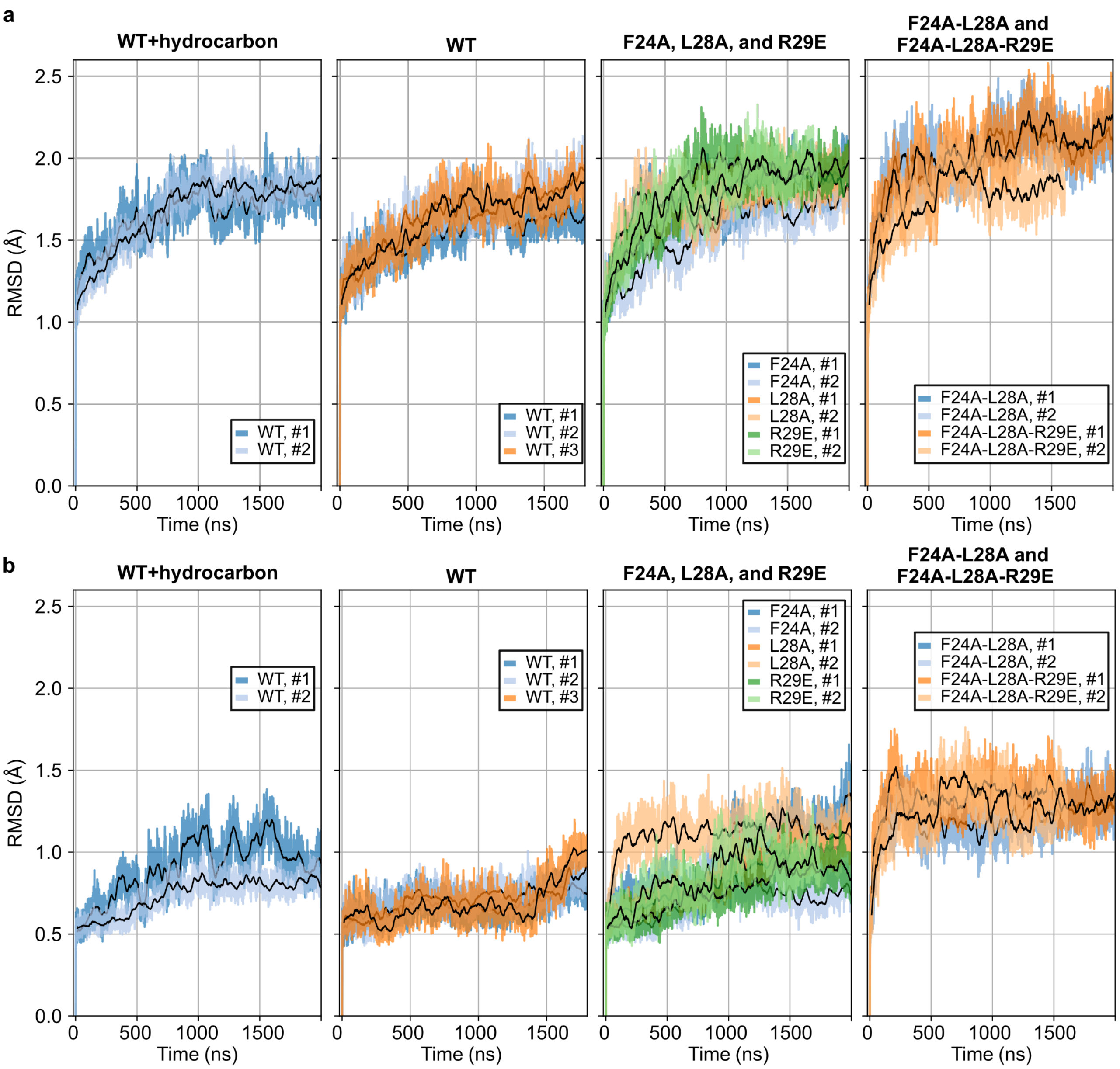

Supplementary Fig. 22 Structural stability of OLPVRII in MD simulations. **a** RMSD time courses calculated for all transmembrane backbone positions of the OLPVRII pentamer using the crystal structure as a reference for wild type simulations with and without bound hydrocarbon chains, and for the F24A, L28A, R29E, F24/L28A, and F24/L28A/R29 mutants without the hydrocarbon chains. For each condition, 2–3 independent production runs have been conducted and the analyses are shown for each replica. Black lines show a running average using a window size of 3 ns. **b** RMSD analysis as in (A) calculated for the backbone positions of the narrowest part of the central ion pore only (residue range 15–29 and 35–50 of TM1 and TM2, respectively). Source data are provided as a Source Data file.

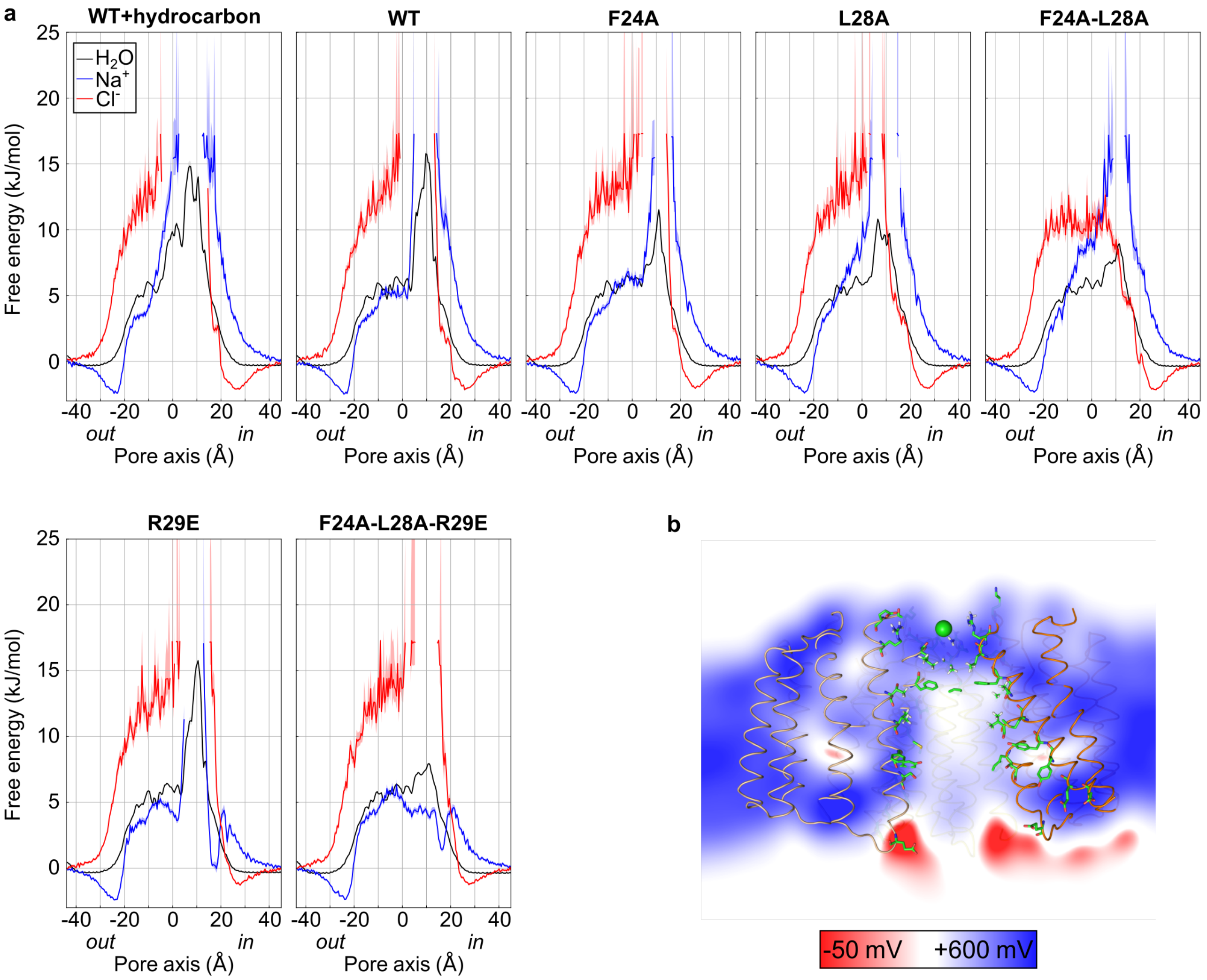

Supplementary Fig. 23 Energetics of ion and water permeation in OLPVRII. **a** Potential of mean force profiles for water, Na^+, and Cl^- permeation along the WT OLPVRII pore with and without bound hydrocarbon chains in the pore, and for the F24A, L28A, R29E, F24/L28A, and F24/L28A/R29E mutants without the hydrocarbon chains. **b** Electrostatic potential distribution calculated from the MD simulations mapped onto a slice through the pore center parallel to the pore axis for wild type OLPVRII (without hydrocarbon chains). Source data are provided as a Source Data file.

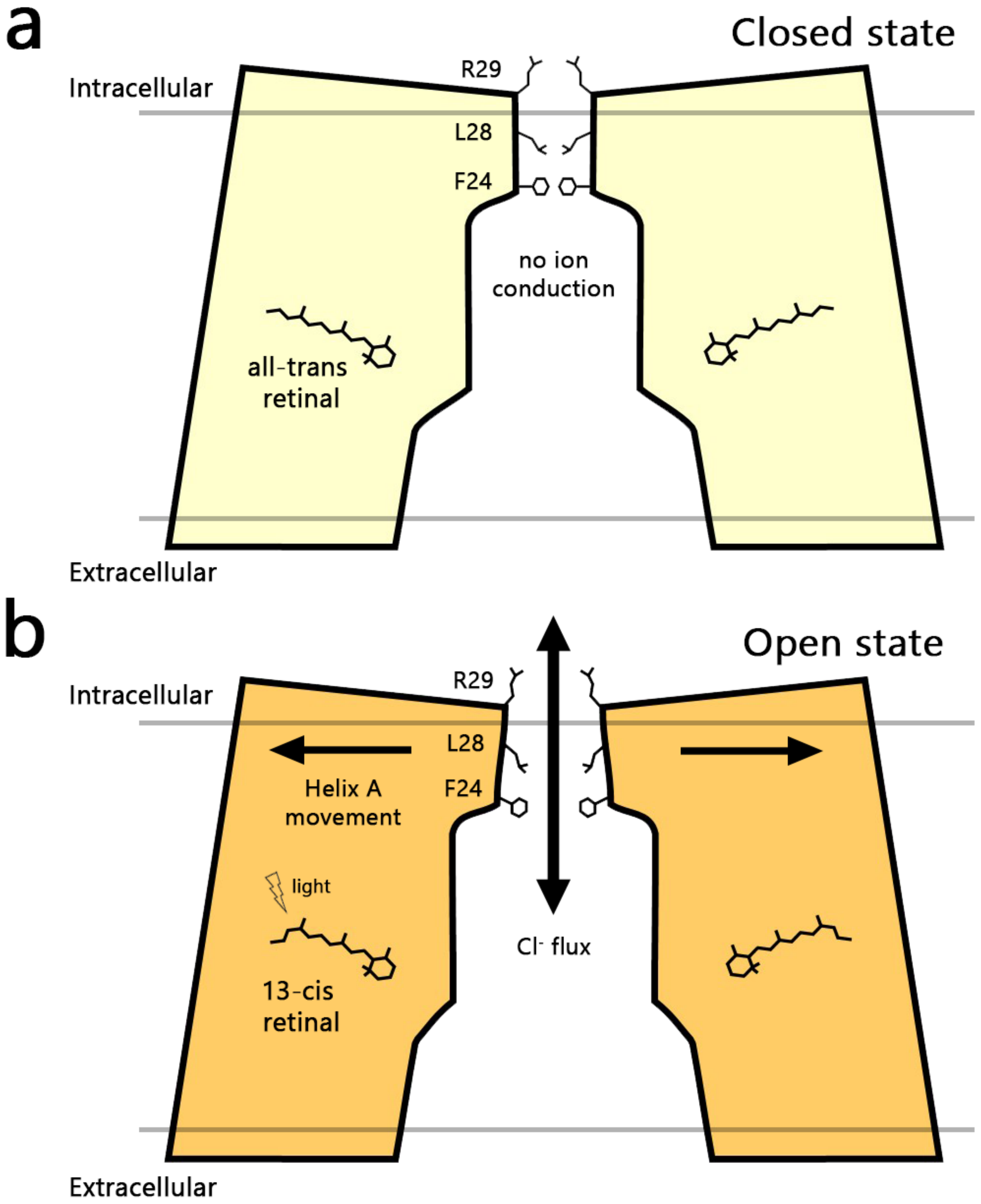

Supplementary Fig. 24 A putative mechanistic scheme of OLPVRII opening/activation. The figure shows the cross section of OLPVRII pentamer through the center of the pore and perpendicular to the membrane plane. A schematic representation of putative switch between closed (upper) and open (lower) states of OLPVRII pentamer is depicted. Possible opening of the channel pore may be induced by outward rotation and shift of helix A, initially caused by retinal cofactor isomerization.

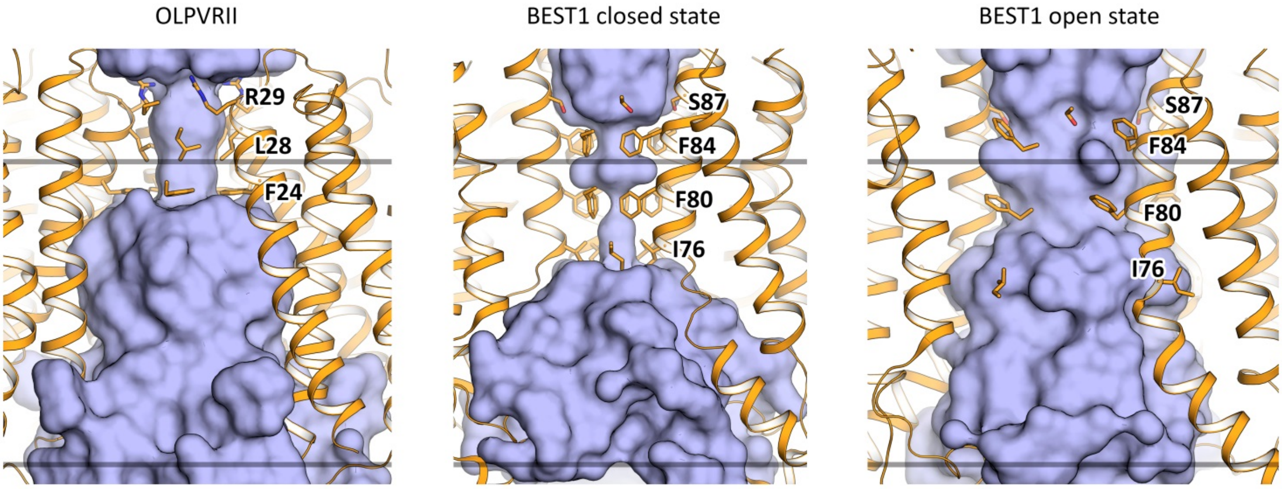

Supplementary Fig. 25 Comparison of transmembrane parts of inner pores of OLPVRII and bestrophin ligand-gated chloride channel (BEST1). As a closed and open states of BEST1 we used models with PDB IDs 6N26 and 6N28, respectively. Membrane core boundaries are shown with gray lines. Residues forming the narrowest parts of the channels are shown with sticks.

**Data collection**	
Space group	P 1 2_1 1
Cell dimensions	
a, *b*, *c* (Å)	79.58, 99.70, 82.96
α, β, γ (°)	90, 117.17, 90
Wavelength (Å)	0.976
Resolution (Å)	41.37-1.90 (1.93-1.90)
R_{merge} (%)	7.2 (142.6)
$I/\sigma I$	10.7 (1.1)
$CC_{1/2}$ (%)	99.9 (38.2)
Completeness	98.9 (98.7)
Multiplicity	4.5 (4.6)
Unique reflections	89527 (4556)
**Refinement**	
Resolution (Å)	41.37-1.90
No. reflections	85,140
No. reflections for R_{free}	4,363
R_{work} / R_{free} (%)	17.1/21.0
No. atoms	
Protein	8513
Retinal	100
Water	217
Lipid fragments	669
B factors (Å^2)	
Protein	38
Retinal	32
Water	48
Lipid fragments	62
R.m.s deviations	
Protein bond lengths (Å)	0.0052
Protein bond angles (°)	0.9873
Ramachandran analysis	
Favored (%)	99.0
Outliers (%)	0.1

Supplementary Table 1 Crystallographic data collection and refinement statistics.

Supplementary Methods

List of primers used in the study

D75N

frw_OLP_219	GTGaATTGGGCAATTACCACC
rev_OLP_194	ATAACGGTTCAGGTTAATTTCTTCG

E42Q

frw_OLP_120	CTGcAgACCTGTATTAGCGTTGTTGCAG
rev_OLP_96	ATTCAGAATATGACGAACGCTTTC

E26A/R36A/W203A

rev_OLP_67	GCTTTCATTTTTGGTACGCAGTGCCGCAATAAAGG
frw_OLP_102	GTTGCCCATATTCTGAATCTGGAAACC
rev_OLP_586	GAAGGCCGCAAAATAGATACCCACAAAGC
frw_OLP_615	TATGCCAAAATCTTTACCGGATCCGGCATTG

List of rhodopsins used for phylogenetic tree

The following rhodopsin sequences were used for sequence alignment and phylogenetic tree construction: NpSR2_sp (P42196, BACS2_NATPH), HsSR2_sp (P71411, BACS2_HALSA), AR2_tr (G1K3Q0, G1K3Q0_ACEAT), LR_tr (Q9HGT7, Q9HGT7_LEPMC), aR-3_sp (P96787, BACR3_HALSD), aR-1_sp (P69051, BACR1_HALS1), aR-2_sp (P29563, BACR2_HALS2), HsBR_sp (P02945,BACR_HALSA), HwBR_sp (Q18DH8, BACR1_HALWD), cR-3_sp (P94854, BACR_HALVA), HmBRI_tr (Q5UXY6, Q5UXY6_HALMA), dR-3_tr (I4DST7, I4DST7_9EURY), MrHR (UPI000362E6CB), SyHR (UPI0002AD07DC), HsHR_sp (P16102, BACH_HALSA), NpHR_tr (Q3ITX1, Q3ITX1_NATPD), ASR_tr (Q8YSC4, Q8YSC4_NOSS1), PoXeR_ref (WP_051881467.1), PspR_gb (EST15450.1), PaR_gb (ADD77066.1), GtCCR1, GtCCR2, GtCCR3, C1C2 (3UG9_A), ChR1_tr (Q93WP2, Q93WP2_CHLRE), ChR2_tr (Q8RUT8, Q8RUT8_CHLRE), GtACR1, GtACR2, XR_tr (Q2S2F8, Q2S2F8_SALRD), TR_tr (H9ZSC3, H9ZSC3_THETH), GR_tr (Q7NP59, Q7NP59_GLOVI), MACR, KR2_tr (N0DKS8, N0DKS8_9FLAO), NM-R3_tr (W8VZW3, W8VZW3_9FLAO), HOT75PR_sp (Q9AFF7, PRRB_PRB02), GPR_sp (Q9F7P4, PRRG_PRB01), Med12PR_tr (Q4PP54, Q4PP54_9BACT), pop_gi (374723690, gb|EHR75770.1), ESR_tr (B1YFV8, B1YFV8_EXIS2), NsXeR_tr (G0QG75, G0QG75_NANS0), HsSR1_sp (P25964, BACS1_HALSA), OLPVRII (F2Y2Z0|F2Y2Z0_9PHYC),

PgVR1_357289866, OlPVR2_322510906, OLPVR1 (ADX06642.1), env_135537302, env_136189461, HKR1_tr (Q6WRU3|Q6WRU3_CHLRE), Rh-GC_tr (A0A060H1D7, A0A060H1D7), Rh-PDE_tr (F2TZN0, F2TZN0_SALR5.
