# Supplementary material for: Unique structure and function of viral rhodopsins
Source: Nat Commun. 2019 Oct 30;10:4939. doi: 10.1038/s41467-019-12718-0 (PMC6821725; doi:10.1038/s41467-019-12718-0)
Supplement: Supplementary file 4 — Description of Additional Supplementary Files [file 41467_2019_12718_MOESM4_ESM.pdf]

Title: Supplementary Data 1

Description: Sequence alignment of viral rhodopsins group II. An alignment of 213 identified complete or partial sequences of group II viral rhodopsins including 5 previously reported.
